# Cardiovascular magnetic resonance imaging of aorto-iliac and ilio-femoral vascular calcifications using proton density-weighted in-phase stack of stars

**DOI:** 10.1186/s12968-018-0479-2

**Published:** 2018-08-06

**Authors:** Ali Serhal, Ioannis Koktzoglou, Pascale Aouad, James C. Carr, Shivraman Giri, Omar Morcos, Robert R. Edelman

**Affiliations:** 1 Radiology, Northwestern Memorial Hospital, Chicago, IL USA; 2 Radiology, Northshore University HealthSystem, Walgreen Building, G534, 2650 Ridge Avenue, Evanston, IL 60201 USA; 3 Radiology, University of Chicago Pritzker School of Medicine, Chicago, IL USA; 4 Siemens Healthineers, Chicago, IL USA; 5 Surgery, Northshore University HealthSystem, Evanston, IL USA

**Keywords:** Vascular calcification, Stack of stars, Magnetic resonance imaging, Quiescent-interval slice-selective, CT angiography, Peripheral arterial disease

## Abstract

**Background:**

Comparing cardiovascular magnetic resonance (CMR) angiography with computed tomography angiography (CTA), a major deficiency has been its inability to reliably image peripheral vascular calcifications that may impact the choice of interventional strategy and influence patient prognosis. Recently, MRI using a proton density-weighted, in-phase stack of stars (PDIP-SOS) technique has proved capable of detecting these calcifications. The goal of the present study was two-fold: (1) to determine whether magnetic field strength impacts the apparent size and conspicuity of ilio-femoral arterial calcifications; and (2) to determine whether the technique can be accurately applied to image aorto-iliac arterial calcifications.

**Main body:**

Two patient cohorts were studied. For the first cohort, ilio-femoral arterial calcifications were imaged at 1.5 Tesla in 20 patients and at 3 Tesla in 12 patients. For the second cohort, aorto-iliac arterial calcifications were imaged in 10 patients at 3 Tesla and one patient at 1.5 Tesla. Qualitative image analysis as well as quantitative analysis using a semi-automated technique were performed using CTA as the reference standard.

Qualitatively, most PDIP-SOS CMR images showed good-to-excellent confidence to detect vascular calcifications, with good-to-excellent inter-reader agreement (κ = 0.67 for ilio-femoral region, *P* < 0.001; κ = 0.80 for aorto-iliac region, *P* < 0.01). There was an overall excellent correlation (*r* = 0.98, P < 0.001) and agreement (intraclass correlation coefficient = 0.97, *P* < 0.001) between PDIP-SOS CMR and CTA measures of calcification volume in both regions, with no overt difference in performance at 1.5 Tesla vs. 3 Tesla for ilio-femoral calcifications. CMR lesion volumes were slightly lower than those measured for CTA.

**Conclusion:**

Using PDIP-SOS CMR, aorto-iliac and ilio-femoral calcifications could be simultaneously evaluated at 3 Tesla in less than six minutes with excellent correlation and agreement to CTA. Our results suggest that PDIP-SOS CMR provides a reliable alternative to CT for pre-interventional evaluation of peripheral vascular calcium burden.

## Background

Both computed tomography angiography (CTA) and cardiovascular magnetic resonance (CMR) angiography are accurate tests for the cross-sectional assessment of peripheral arterial disease (PAD) [1, 2]. Compared with CMR angiography, CTA offers higher speed, lower cost, and fewer drawbacks overall. Nonetheless, peripheral CMR angiography remains in widespread use, in part because it avoids exposure to ionizing radiation and, given that patients with PAD often suffer from poor renal function [3], offers the option of imaging without an exogenous contrast agent [4].

In diabetic patients, CMR angiography is advantageous compared with CTA because of the high prevalence of peripheral vascular calcifications [5]. With CTA, these calcifications confound the evaluation of small caliber vessels due to blooming artifact that obscures the vessel lumen [6]. Since vascular calcifications are unapparent and do not cause image artifacts with CMR angiography, it is often preferred to CTA in this patient group. However, important information deriving from the presence of vascular calcifications, including the impact on interventional strategy and patient prognosis, is then lost [7–10]. For instance, dense arterial wall calcifications should be avoided when choosing a percutaneous access site, and their presence is a major determinant of failure for percutaneous endovascular aneurysm repair [10].

The inability to image vascular calcifications remains an important and unaddressed deficiency of CMR angiography. For instance, in the largest clinical trial to evaluate the association of plaque characteristics with functional performance in patients with PAD, investigators were unable to reliably identify vascular calcifications [11]. The only way to recover this information would be to perform an additional CT scan, which is inconvenient and costly.

Vascular calcifications have negligible signal intensity with standard CMR pulse sequences due to low free water concentration and short T2* [12]. While this property makes them difficult to visualize with CMR angiography, a recent study demonstrated the feasibility of using “neutral contrast” 3D gradient-echo techniques to depict peripheral vascular calcifications on minimum intensity projection CMR images [13]. However, a major unknown with using CMR to image vascular calcifications is the potential impact of field strength on apparent lesion volumes. Just as calcifications can show blooming artifact with CT, diamagnetic susceptibility and T2* effects have the potential to cause field strength-dependent blooming artifact with CMR. Vascular calcifications are strongly diamagnetic - as much as ten times that of the vessel wall – which results in field strength-dependent phase shifts [14]. Moreover, the signal-to-noise ratio (SNR), R2*, chemical shift and off-resonance effects all increase with field strength.

Another unknown is whether CMR can reliably image vascular calcifications in the abdomen and pelvis. In these regions, respiratory motion as well as presence of peristalsing, air-containing bowel loops have the potential to cause blurring and off-resonance effects, thereby obscuring vessel calcifications.

To address these concerns, the goals of the present study were two-fold: (1) to determine whether magnetic field strength impacts the apparent size and conspicuity of ilio-femoral arterial calcifications; and (2) to determine whether the technique can be applied to accurately image aorto-iliac arterial calcifications.

## Methods

This prospective study was approved by the Institutional Review Boards of two academic centers and informed consent was obtained. Two cohorts of patients were studied. For the first cohort, 32 patients (52–84 years, 10 female) were consecutively recruited from those in whom CTA performed within the preceding three months demonstrated vascular calcifications in at least one ilio-femoral vessel segment. At one institution, 20 patients were imaged using a 1.5 Tesla MAGNETOM Aera scanner (Siemens Healthineers, Erlangen, German), while at the other institution 12 patients were imaged using a 3.0 Tesla MAGNETOM Skyra Fit scanner (Siemens Healthineers). For the second cohort, 11 patients (57–83 years, 2 female) were recruited from those in whom CTA demonstrated vascular calcifications in the region of the aorto-iliac bifurcation; ten patients in this cohort were imaged at 3 Tesla, while one patient was imaged at 1.5 Tesla.

Nonenhanced quiescent-interval slice-selective (QISS) CMR angiography [15] of the peripheral arteries was used as the scout for positioning the proton density-weighted, in-phase stack-of-stars (PDIP-SOS) spoiled gradient-echo pulse sequence. This sequence provides nearly isotropic images in which muscle, fat, and intravascular signal show intermediate signal intensity, whereas vascular calcifications appear uniformly dark [16]. Unlike a Cartesian 3D acquisition, in which chemical shift artifacts at fat/water interfaces appear as discrete dark lines that can obscure or be confounded with vascular calcifications in minimum intensity projections, these interfaces are less distinct with the in-phase stack-of-stars radial k-space trajectory so that they are invisible in the projection images.

For the first cohort of patients, the PDIP-SOS sequence used a legacy encoding scheme whereby all radial views were collected in rapid sequence as a single shot. For the second cohort of patients, the k-space sampling strategy was updated so that all slice partitions, rather than radial views, were collected in rapid sequence.

PDIP-SOS images were acquired in an oblique coronal plane. Body and peripheral phased array coils were used for signal reception. There were some differences in the imaging parameters between the two magnetic field strengths, due primarily to the in-phase echo time at 1.5 Tesla being twice the value at 3 Tesla and the use of a lower sampling bandwidth to improve the SNR, in turn lengthening the repetition time. Consequently, some compromises in spatial resolution were needed at 1.5 Tesla to avoid increasing scan time and to maintain adequate SNR despite the lower field strength. Slice thickness was 1.3-mm at 1.5 Tesla and 1.0-mm at 3 Tesla, with 128 reconstructed slices per 3D slab and in-plane resolution at both field strengths of 1.0-mm × 1.0-mm prior to interpolation. For the second patient cohort using the updated k-space sampling strategy, PDIP-SOS CMR encompassing both the aorto-iliac and ilio-femoral regions was acquired with 1200 radial views in a scan time of 5 min 49 s. The PDIP-SOS images were processed into thin (4 to 15-mm) minimum intensity projections for qualitative display of the vascular calcifications.

The CTA studies were acquired on three different scanners: SOMATOM Force, SOMATOM Definition AS and SOMATOM Definition (Siemens Healthineers, Forchheim, Germany) with standard technique using either iterative reconstruction or filtered back projection according to clinical routine. Typical slice thickness was on the order of 0.6-mm to 1-mm. kVp was selected according to the pre-determined body mass index-based institutional protocol.

### Quantitative image analysis

Quantification of the calcification volumes was done using in-house software executed within ImageJ (version 1.51p, National Institutes of Health, Bethesda, Maryland, USA). For analysis of the ilio-femoral region, on each lower limb extremity, two segments of the femoral artery were analyzed: the proximal segment which covered 5 cm of the common femoral artery immediately above the femoral bifurcation, and the distal segment which covered 5 cm of the superficial femoral artery immediately below the bifurcation. For analysis of the aorto-iliac region, five segments of the aorta and iliac vessels were analyzed: the most distal 5 cm segment of the aorta, the left and right common iliac arteries, and the left and right external iliac arteries. The segmentation algorithm volumetrically segmented the calcifications using thresholds based on Hounsfield units for CTA images and on signal intensity for CMR images. Using this algorithm, we have previously found that a threshold of 560 Hounsfield units was optimal for distinguishing calcifications on CTA from contrast-enhanced lumen. For CMR images of the ilio-femoral region, voxels having a signal intensity more than three standard deviations below the mean were classified as calcifications. A CMR threshold of two (instead of three) standard deviations was used in the aorto-iliac region due to slightly increased background signal variability in this region from bowel and respiratory motion. Bicubic interpolation was used to scale all CMR and CTA data to 0.5 mm isotropic resolution before measurement of calcification volume.

### Qualitative image analysis

Two cardiovascular radiology fellows (AS, PA), blinded to clinical information, qualitatively reviewed source images and multi-planar reformats for both CTA and CMR. CTA images were taken as a reference and the PDIP-SOS CMR images were analyzed for degree of confidence to detect calcification, matching size, shape and location of calcification with the CTA images. Images were scored using a 5-point Likert scale as: 1- very poor, 2- poor, 3- fair, 4- good, 5- excellent.

### Data and statistical analysis

Correlation between volume of vascular calcification shown by PDIP-SOS and CTA was assessed by linear regression and Pearson’s correlation analysis (with correlation coefficient, *r*). Agreement of vascular calcification volume was assessed by intraclass correlation coefficient (ICC). Sub-analyses of data in the ilio-femoral region were performed by stratifying according to magnetic field strength (1.5 Tesla and 3 Tesla), and severity of calcification (none or mild, moderate, severe) based on calcification volume measures on CTA. Group 1 included patients with no or mild vascular calcification (volume 0–99 mm^3^), group 2 included patients with moderate vascular calcification (volume 100–299 mm^3^), and group 3 included patients with severe vascular calcification (volume ≥ 300 mm^3^). Weighted Cohen’s kappa (κ) coefficient was used to assess inter-rater agreement of qualitative scores. Pearson correlation (r), ICC, and κ coefficients were interpreted as follows: < 0.40 – poor, 0.40–0.59 – fair, 0.60–0.74 – good, ≥0.75 – excellent [17]. Bland-Altman analysis was used to compute mean bias (MR volume minus CTA volume) and 95% limits of agreement. Statistical analyses were performed in R software (version 3.4.2, R Foundation for Statistical Computing, Vienna, Austria).

## Results

### Cohort 1 (Ilio-femoral vessels)

Of 128 ilio-femoral vessel segments available for analysis, 6 were excluded (4 due to magnetic susceptibility artifact from hip prosthesis or stent and 2 due to artifact relating to recent bypass graft), leaving a total of 122 vessel segments that were analyzed.

**Fig. 1:**
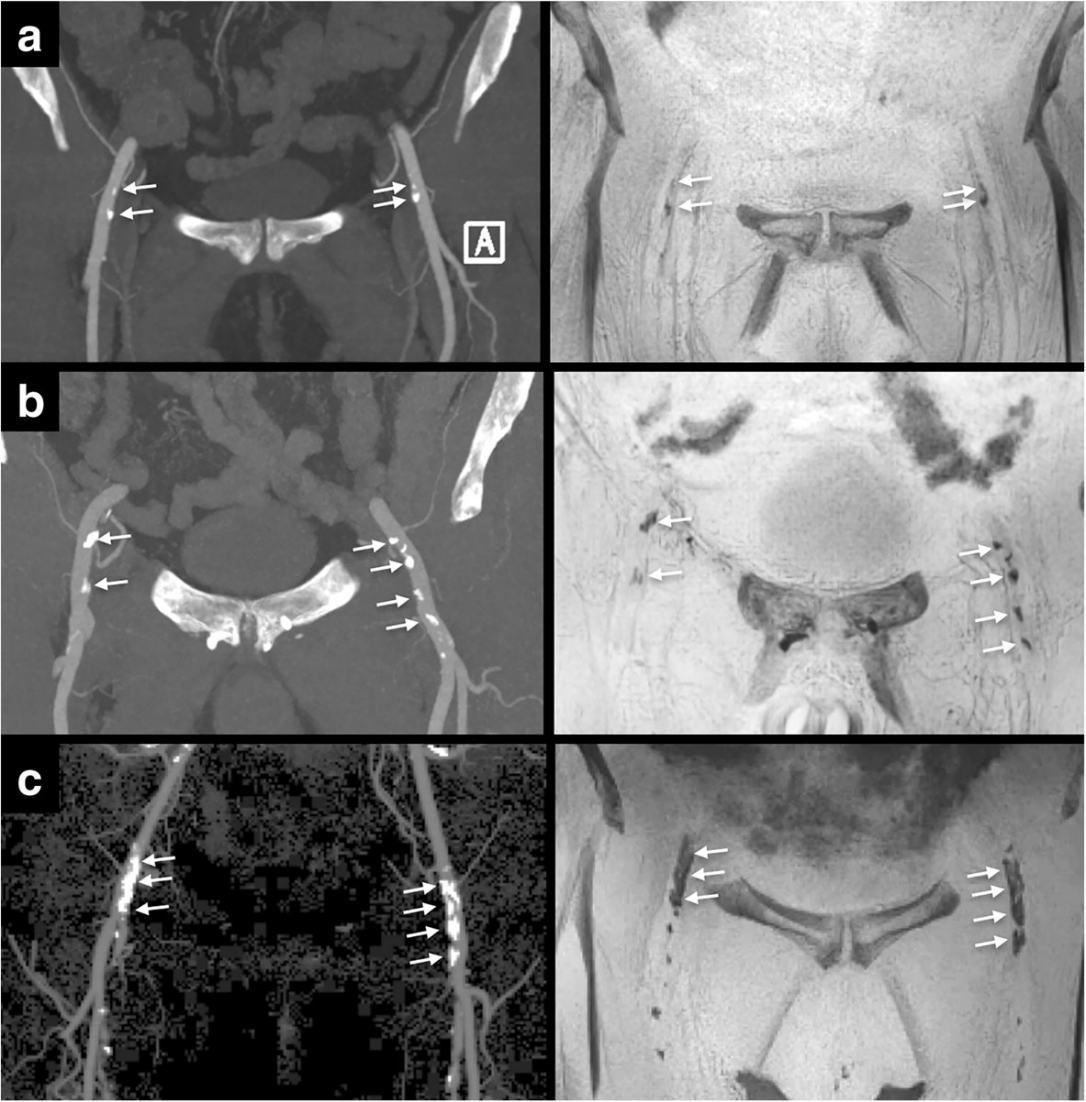
Examples of PDIP-SOS CMR (right) in comparison to CTA (left) for: (**a**) 66-year-old female imaged at 1.5 Tesla showing mild vascular calcifications (Group 1), (**b**) 80-year-old male imaged at 1.5 Tesla showing moderate vascular calcifications (Group 2), and (**c**) 68-year-old female imaged at 3 Tesla showing severe vascular calcifications (Group 3). Vascular calcifications (arrows) appear dark with PDIP-CMR (presented as thin minimum intensity projections), and bright with CTA (presented as thin maximum intensity projections). There is excellent correlation between CMR and CTA irrespective of lesion severity

Qualitatively, PDIP-SOS CMR images showed good-to-excellent confidence to detect ilio-femoral vascular calcifications with a mean score of 4.1 (range 3–5) for the first reader and 4.3 (range 3–5) for the second reader. Inter-rater agreement was good (κ=0.67, 95% confidence interval (CI): 0.54–0.80, *P* < 0.001). The size, shape and location of the calcifications by PDIP-SOS CMR matched CTA findings in all patients regardless of lesion volume (Fig. 1).

**Fig. 2:**
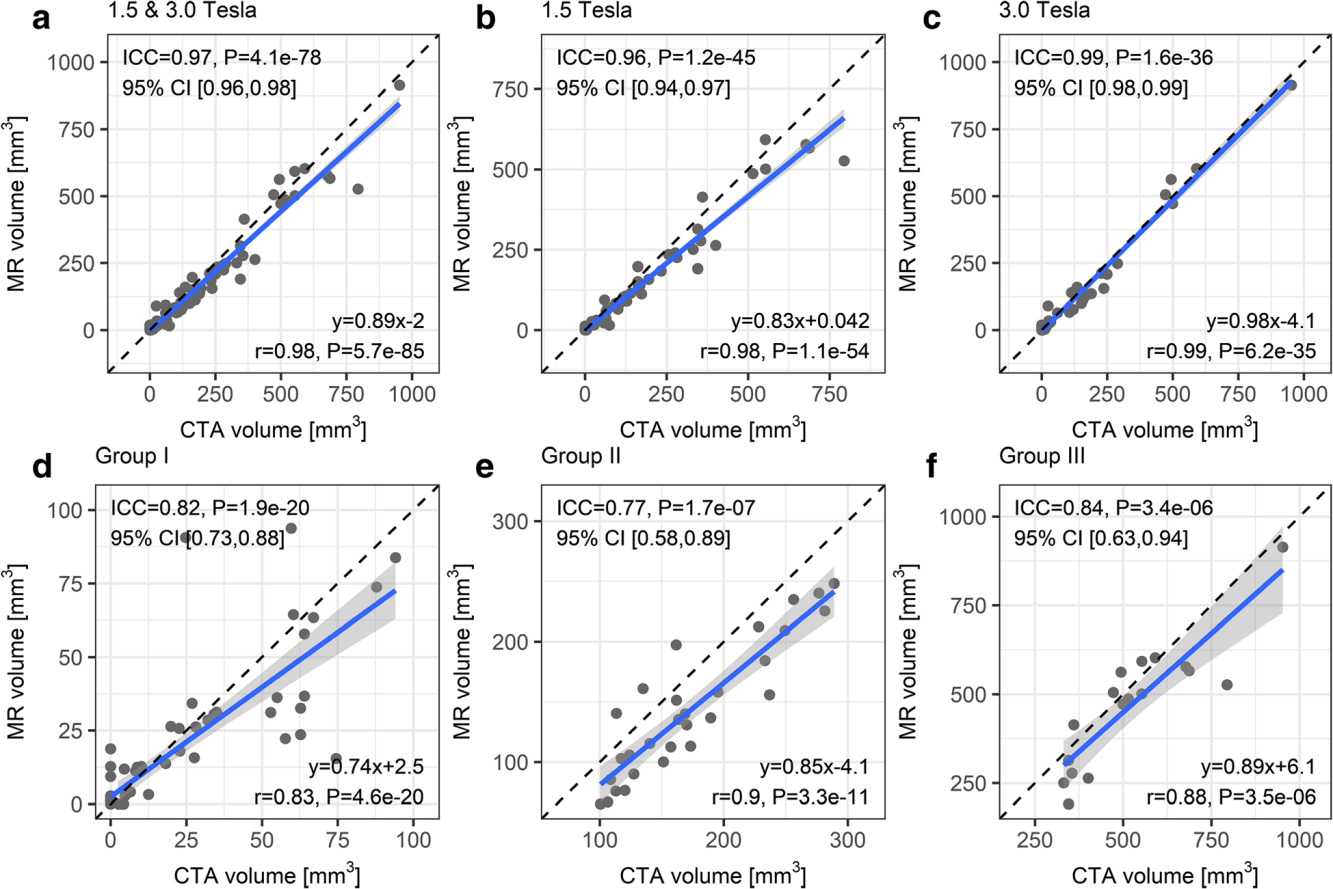
Scatter plots of ilio-femoral calcification volume as measured by PDIP-SOS CMR versus CTA at (**a**) both magnetic field strengths, (**b**) 1.5 T, and (**c**) 3 T, as well as by severity of calcification (**d)-(f**). Linear regression equations are shown at bottom right. Solid lines and gray areas show the lines of best fit and the 95% confidence intervals, respectively. Dashed lines are lines of unity

Figure 2 shows scatter plots of calcification volume in the ilio-femoral region stratified by magnetic field strength and severity of calcification. There was an overall excellent correlation (*r* = 0.98, 95% CI: 0.97–0.99, *P* < 0.001) and agreement (ICC = 0.97, 95% CI: 0.96–0.98, *P* < 0.001) between PDIP-SOS CMR and CTA measures of calcification volume (Fig. 2a). The average volume was 124 mm^3^ (range 0–953 mm^3^) by CTA and 109 mm^3^ (range 0–914 mm^3^) by CMR. When comparing the results of CMR exams done on 1.5 Tesla and 3 Tesla, the correlation coefficients were *r* = 0.98 (95% CI: 0.97–0.99, *P* < 0.001) and 0.99 (95% CI: 0.98–0.99, *P* < 0.001), while ICC values were 0.96 (95% CI: 0.94–0.97, *P* < 0.001) and 0.99 (95% CI: 0.98–0.99, *P* < 0.001), respectively (Figs. 2b and c). Bland-Altman mean biases and 95% limits of agreement (CMR minus CTA calcification volume) at both magnetic field strengths, 1.5 Tesla, and 3 Tesla were − 15.7 [− 95.2, 63.8] mm^3^, − 20.3 [− 107.9, 67.3] mm^3^, and − 7.0 [− 65.4, 51.4] mm^3^, respectively.

The Pearson correlation coefficients of calcification volume for patients with no-to-mild, moderate and severe vascular calcifications were *r* = 0.83 (95% CI: 0.74–0.89), 0.90 (95% CI: 0.80–0.95), and 0.88 (95% CI: 0.69–0.96) (*P* < 0.001 for all), respectively (Figs. 2d-f). Corresponding ICC values for agreement in these three strata remained in the excellent range and were 0.82 (95% CI: 0.73–0.88), 0.77 (95% CI: 0.58–0.89), and 0.84 (95% CI: 0.63–0.94) (*P* < 0.001 for all), respectively. Bland-Altman mean biases [95% limits of agreement] for patients with no-to-mild, moderate, and severe vascular calcifications were − 1.7 [− 30.0, 26.6] mm^3^, − 30.3 [− 80.5, 19.9] mm^3^, and − 53.4 [− 223.1, 116.3] mm^3^, respectively.

### Cohort 2 (aorto-iliac vessels)

**Fig. 3:**
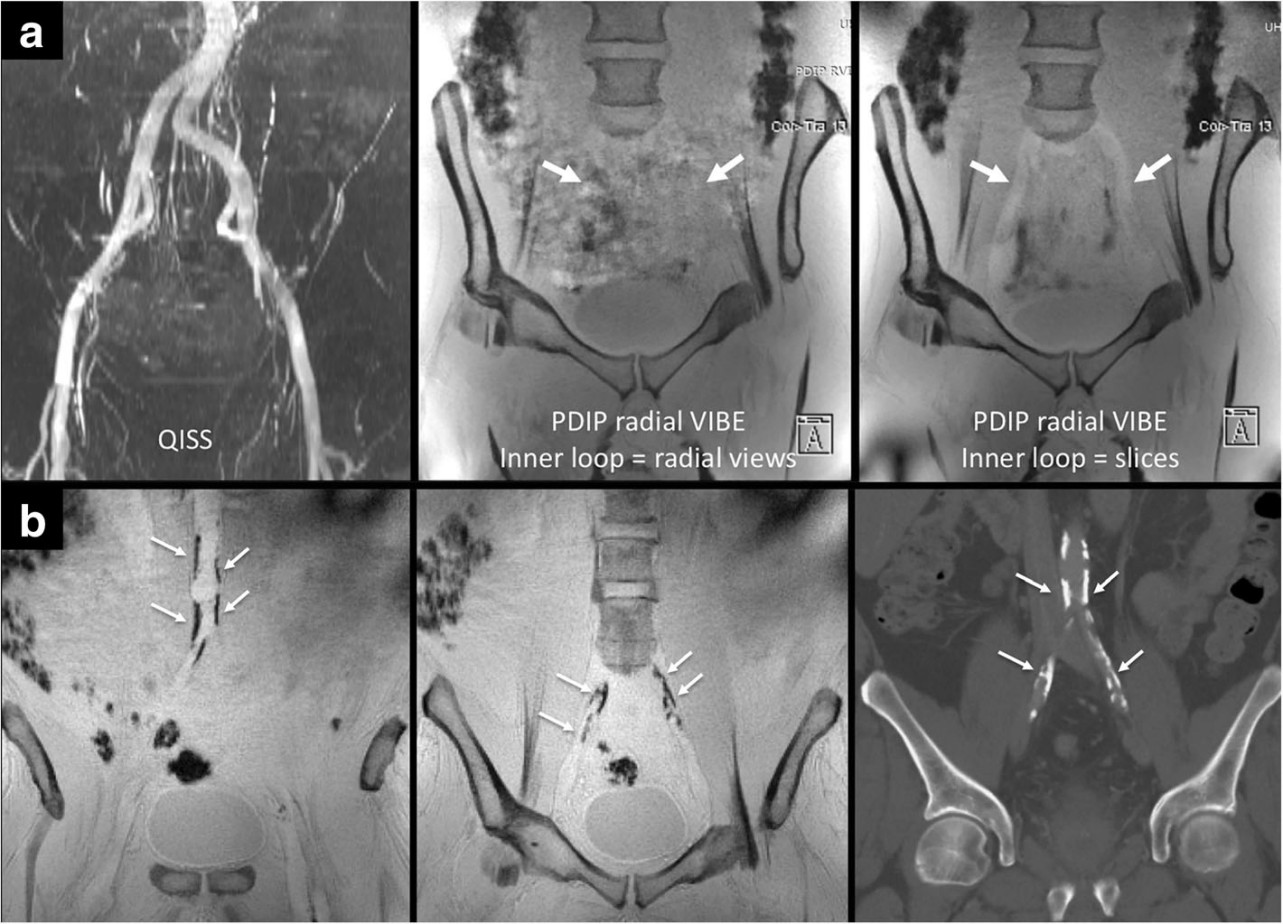
Evaluation of the aorto-iliac vessels using PDIP-SOS CMR. (**a**) Healthy volunteer. Left: QISS nonenhanced CMR angiogram used for positioning the stack of stars imaging slab. Middle: PDIP-SOS CMR using the legacy k-space encoding approach of acquiring all radial views in rapid sequence. There are severe ghost artifacts arising from centrally-located bowel loops that obscure the iliac vessels (arrows). Right: Identical PDIP-SOS acquisition except that all slice partitions (instead of radial views) were acquired in rapid sequence. The bowel-related artifacts are eliminated, so that the iliac vessels are well shown (arrows). Note that both the aorto-iliac and ilio-femoral regions are encompassed in the field of view, allowing simultaneous assessment of both regions. (**b**) Patient with aorto-iliac calcifications. Left, middle: 4-mm thick minimum intensity projections from PDIP-SOS CMR clearly depict vascular calcifications involving the distal aorta and proximal iliac vessels. Right: Thin-slab maximum intensity coronal projection from the CT shows excellent correspondence with the PDIP-SOS CMR

Initial studies demonstrated that the order in which the slice partitions and radial views were collected, while largely irrelevant for the ilio-femoral vessels, had a profound effect on image quality for the aorto-iliac vessels. Acquisitions using a legacy k-space encoding strategy in which the radial views were collected in rapid sequence as a single shot typically showed extensive artifacts relating to air-containing bowel loops which often obscured the distal aorta and pelvic arteries. In contrast, these artifacts were largely absent when the slice partitions were collected in rapid sequence as a single shot allowing for unambiguous identification of vascular calcifications (Fig. 3).

A total of 55 aorto-iliac vessel segments were analyzed, and none were excluded. Qualitatively, most PDIP-SOS CMR images showed good-to-excellent confidence to detect aorto-iliac vascular calcifications with a mean score of 4.1 (range 3–5) for the first reader and 4.0 (range 3–5) for the second reader. Inter-rater agreement was excellent (κ=0.80, 95% confidence interval (CI): 0.57–1.00, *P* < 0.01).

**Fig. 4:**
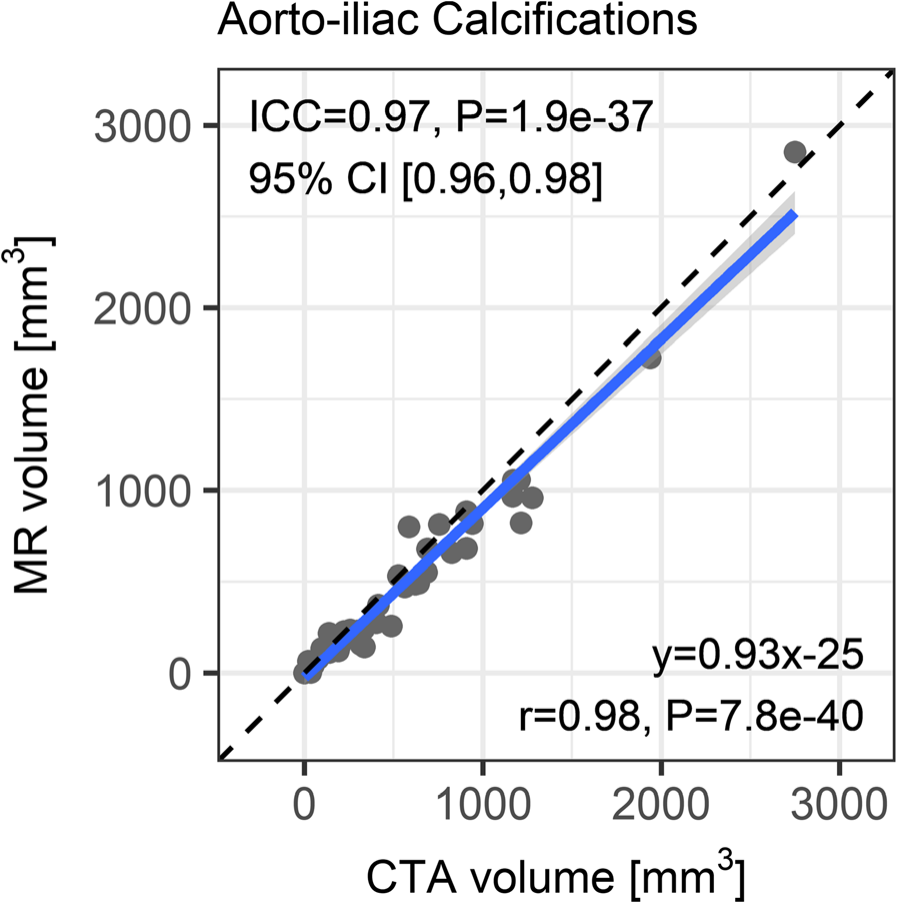
Scatter plot of aorto-iliac calcification volume as measured by PDIP-SOS CMR versus CTA. Linear regression equation is shown at bottom right. The solid line and gray area show the line of best fit and the 95% confidence interval, respectively. Dashed line shows the line of unity

Figure 4 shows a scatter plot of calcification volume in the aorto-iliac region. There was an overall excellent correlation (*r* = 0.98, 95% CI: 0.97–0.99, *P* < 0.001) and agreement (ICC = 0.97, 95% CI: 0.96–0.98, *P* < 0.001) between PDIP-SOS CMR and CTA measures of calcification volume. The average volume was 450 mm^3^ (range 0–2750 mm^3^) by CTA and 391 mm^3^ (range 0–2854 mm^3^) by CMR. The Bland-Altman (CMR minus CTA calcification volume) mean bias was − 58.5 mm^3^, while the 95% limits of agreement were [− 261.8, 144.7] mm^3^.

## Discussion

Prior work has shown that CMR using a PDIP-SOS pulse sequence is accurate for ilio-femoral vascular calcifications [16]. The present work differs in three significant ways from this prior work. First, the present study was sufficiently powered to examine the impact of magnetic field strength (1.5 Tesla vs. 3 Tesla) on sequence performance and apparent lesion size for ilio-femoral calcifications. Second, we used a new motion-resistant k-space trajectory to additionally enable evaluation of aorto-iliac calcifications, which was not possible with earlier versions of the technique due to excessive respiratory and bowel motion artifacts. Third, a new cohort of patients with PAD was used for the present study with no overlap to the prior study.

Our results indicate that CMR using a PDIP-SOS pulse sequence depicts ilio-femoral and aorto-iliac calcifications with excellent correlation (*r* ≥ 0.98) and agreement (ICC ≥ 0.97) to CTA over a wide range of lesion volumes. The correlation and agreement to CTA for ilio-femoral calcifications were similar at 1.5 Tesla and 3 Tesla, suggesting that lesion volumes as measured by PDIP-SOS CMR are not substantially dependent on magnetic field strength. Regardless of field strength, in no case did CMR miss large calcifications that might adversely impact the outcome of a percutaneous procedure, nor falsely suggest significant calcifications where none existed in the CTA.

Given that CMR is generally considered to be insensitive to vascular calcifications, it is notable that the PDIP-SOS imaging technique could detect calcifications over a wide range of lesion volumes, including small vascular calcifications that spanned only a few pixels. However, the use of a very small voxel (e.g. ~ 1 mm^3^ in the current study) and adequate SNR are essential for confidently detecting small lesions.

The stack of stars k-space trajectory has the benefit of being less sensitive to artifacts from respiratory motion than a Cartesian 3D acquisition, due to extensive oversampling of the center of k-space [18]. Nonetheless, we found that our legacy k-space encoding approach for PDIP-SOS, in which all radial views were acquired in rapid sequence as a single shot, often showed severe image artifacts in the pelvic and abdominal regions due to respiratory and peristaltic motion of bowel loops. These bowel-related artifacts often obscured nearby vascular calcifications. In order to overcome these artifacts, we modified the k-space sampling strategy to acquire all slice partitions in rapid sequence rather than radial views. A similar sampling strategy has previously been reported to reduce respiratory motion artifact in the upper abdomen [19], and we found it to be highly effective at suppressing artifacts from bowel motion in the lower abdomen and pelvis as well. With this updated PDIP-SOS CMR technique, it is now possible to simultaneously and accurately assess vascular calcifications in both the aorto-iliac and ilio-femoral regions with a single, < 6-min oblique coronal acquisition.

Across all patients at both magnetic field strengths, CMR slightly underestimated calcification volumes (linear regression slopes of ~ 0.89 and ~ 0.93 for ilio-femoral and aorto-iliac regions) compared with CTA. This discrepancy might be explained by the fact that blooming artifact artifactually enlarges dense calcifications on CT images [20]. Alternatively, it is conceivable that the surface regions of a calcification contain mobile water spins that produce detectable signal intensity, which would decrease their apparent volume with PDIP-SOS CMR.

A limitation of our study was that contrast-enhanced CTA was used as a reference standard, whereas non-contrast CT would be preferred. However, at the two institutions used for this study, non-contrast CT series were acquired at 3 to 5-mm slice thickness, too thick for volumetric evaluation, compared with 0.6 to 1-mm slice thickness for CTA. Also, PDIP-SOS CMR scan times were substantially longer than is the case with CT. Scan time can be greatly reduced (e.g. to 30 s or less) by acquiring thicker slices and using fewer radial views at the expense of more partial volume averaging, decreased SNR and increased radial streak artifacts. While this accelerated approach would reduce sensitivity for very small calcifications, it is unlikely that detection of bulky, clinically significant calcifications would be adversely affected. Finally, our study demonstrated that imaging of both aorto-iliac and ilio-femoral calcifications is accurate at 3 Tesla. While imaging of ilio-femoral calcifications is also accurate at 1.5 Tesla, the study was underpowered (*n* = 1) at this field strength to draw any conclusions about aorto-iliac calcifications.

## Conclusion

Using PDIP-SOS CMR, aorto-iliac and ilio-femoral calcifications could be simultaneously evaluated at 3 Tesla in less than six minutes with excellent correlation and agreement to CTA. Lesion size was not substantially affected by magnetic field strength. Our results suggest that PDIP-SOS CMR provides a reliable alternative to CT for pre-interventional evaluation of peripheral vascular calcium burden.

## Abbreviations

CMR: cardiovascular magnetic resonance
CT: computed tomography
CTA: computed tomographic angiography
PAD: peripheral arterial disease
PDIP-SOS: proton density-weighted, in-phase stack-of-stars
QISS: Quiescent-Interval Slice-Selective
SNR: signal-to-noise ratio
